# Lessons learned in Liberia: preliminary examination of the psychometric properties of trust and teamwork among maternal healthcare workers

**DOI:** 10.1186/1472-6963-13-134

**Published:** 2013-04-11

**Authors:** Jody R Lori, Michelle L Munro, Jennifer E Moore, Jessica Fladger

**Affiliations:** 1 Division of Health Promotion and Risk Reduction, University of Michigan, School of Nursing, 400 N. Ingalls, Room 3352, Ann Arbor, MI 48109, USA; 2 Division of Nursing Business and Health Systems, University of Michigan, School of Nursing, 400 N. Ingalls, Room 4170, Ann Arbor, MI 48109, USA

**Keywords:** Trust, Teamwork, Traditional birth attendants, Certified midwives, Psychometric testing

## Abstract

**Background:**

Post-conflict Liberia has one of the fastest growing populations on the continent and one of the highest maternal mortality rates among the world. However, in the rural regions, less than half of all births are attended by a skilled birth attendant. There is a need to evaluate the relationship between trained traditional healthcare providers and skilled birth attendants to improve maternal health outcomes. This evaluation must also take into consideration the needs and desires of the patients. The purpose of this pilot study was to establish the validity and reliability of a survey tool to evaluate trust and teamwork in the working relationships between trained traditional midwives and certified midwives in a post-conflict country.

**Methods:**

A previously established scale, the *Trust and Teambuilding Scale*, was used with non- and low-literate trained traditional midwives (n=48) in rural Liberia to evaluate trust and teamwork with certified midwives in their communities. Initial results indicated that the scale and response keys were culturally inadequate for this population. A revised version of the scale, the *Trust and Teamwork Scale – Liberia*, was created and administered to an additional group of non- and low-literate, trained traditional midwives (n=42). Exploratory factor analysis using Mplus for dichotomous variables was used to determine the psychometric properties of the revised scale and was then confirmed with the full sample (n=90). Additional analyses included contrast validity, convergent validity, and Kuder-Richardson reliability.

**Results:**

Exploratory factor analysis revealed two factors in the revised *Trust and Teamwork Scale – Liberia*. These two factors, labeled trust and teamwork, included eleven of the original eighteen items used in the *Trust and Teamwork Scale* and demonstrated contrast and convergent validity and adequate reliability.

**Conclusions:**

The revised scale is suitable for use with non- and low-literate, trained traditional midwives in rural Liberia. Continued cross-cultural validation of tools is essential to ensure scale adequacy across populations. Future work should continue to evaluate the use of the *Trust and Teamwork Scale – Liberia* across cultures and additional work is needed to confirm the factor structure*.*

## 

Healthcare systems are failing women in Africa. Sub-Saharan Africa suffers from the worst maternal mortality rates in the world with over 500/100,000 live births resulting in maternal death [1]. Liberia has one of the highest maternal mortality rates among the world with a rate of 994/100,000 live births [2]. In 2010, Liberia was ranked 7th highest for maternal mortality worldwide with an estimated 1,200 maternal deaths annually [1].

Bream and Buor [3] attribute these high rates of maternal mortality to higher total fertility rates and maternal illiteracy in sub-Saharan Africa. Their analysis of existing data from the World Health Organization revealed the use of skilled birth attendants (certified midwives [CMs], doctors, and nurses who are trained in the skills to manage normal pregnancy, childbirth, postpartum, and referral) was directly related to a decrease in maternal mortality. They concluded that traditional community norms surrounding childbirth, including the use of unskilled personnel such as trained traditional midwives (TTMs), have negative consequences on maternal survival rates [3].

While some believe TTMs hinder efforts to improve maternal outcomes, it has also been found that when skilled birth attendants and TTMs work together, as opposed to TTMs working independently, maternal outcomes improve [4]. A recent meta-analysis of women receiving care from TTMs found improved maternal and neonatal outcomes when the TTMs received the following: 1) initial and continuous training; 2) ongoing support from skilled birth attendants; 3) formal linkages with healthcare institutions providing resources (e.g., clean birth kits and resuscitation equipment); and 4) institutions that served as a referral pathway [5].

Women often use traditional birth attendants, such as TTMs, for care during pregnancy due to convenience; including proximity and cost. As demonstrated in post-conflict Sierra Leone, women also seek care from traditional birth attendants because of their leadership status and vast experience in the community. Women also have an established trust in the compassionate care provided by traditional birth attendants [6].

As we continue the quest to reach the Millennium Development Goals of reducing maternal and neonatal mortality, it is essential we consider who is available at the community level to childbearing women. The relationship between skilled birth attendants, such as CMs and TTMs may offer insight into developing a collaborative approach to reducing maternal mortality rates while considering the needs and wants of this vulnerable population. The purpose of this pilot study was to establish the validity and reliability of a survey tool to evaluate trust and teamwork in the working relationships between TTMs and CMs in a post-conflict country.

## Background

Liberia has one of the fastest growing populations on the continent with an estimated growth rate of 2.6 and a total fertility rate of 5.2% [2]. After a devastating 14 year civil war in which rebel forces destroyed hospitals, clinics, electricity, and other essential resources, the country has been left with some of the poorest health statistics on the continent. Additionally, it is estimated that two thirds of Liberian women were subjected to gender-based violence during the civil war including sexual assault, rape, and murder contributing to poor physical and mental health outcomes [7].

Liberian women often defer to their husbands or elders when making decisions about their healthcare needs. Many women prefer to give birth in their own community with providers they know and trust, following community norms, rather than seeking care for life-threatening conditions [8]. Lori and Boyle [8] found women utilize multiple types of care providers, both skilled and traditional, during pregnancy and childbirth. This is consistent with the findings from Kruk and colleagues [9] who reported Liberians use both types of providers (TTMs and CMs) as complements to one another, not substitutes, for their care. Importantly, Lori and Boyle [8] reported Liberian women have confidence and trust in TTMs, preferring to deliver with them because they receive more care and attention. They also cite poor relations with professional staff at health facilities as another reason they favor the care of the TTMs [8].

In 2007, 46% of all births in Liberia were attended by a skilled birth attendant, with the lowest rates of skilled birth attendance in the northern central region of the country [10]. There are critical shortages of health professionals in Liberia [11] with the rural poor severely compromised in their ability to access healthcare [12]. Mothers and children in Liberia suffer the greatest morbidity and mortality related to the shortage of healthcare providers [12]. In 2005, post-conflict Liberia had only one doctor and 27 nurses or midwives per 100,000 people [13] making it difficult to access skilled care. While targeted programs work to scale up skilled birth attendants; capitalizing on the present skill mix of both skilled professional and traditional providers could contribute immediately to improved maternal and newborn health.

### Literature review

Within sub-Saharan countries the healthcare system places a higher value on CMs than unskilled providers, with TTMs often viewed as archaic, uneducated support persons [14]. Unfortunately, this perspective fails to recognize the important role of community norms and the acceptance of TTMs as an integral part of the childbirth experience for women. A qualitative study of 84 TTMs in Kenya by Dietsch [4], explored what it means to be a traditional midwife. A major theme of the study included the relationship between the skilled birth attendant and the TTM. The findings emphasized the unrecognized abuse inflicted upon TTMs by skilled birth attendants. According to findings by Dietsch, skilled birth attendants have been known to “exert power in a destructive, de-humanizing, and abusive way over women and TTMs” [4], p.2]. As described by the TTMs, very few midwifery relationships were based on mutual respect and collaborative practice. The TTMs also perceived skilled birth attendants blamed them for labor and birth complications and would intentionally deny care to women under their care.

Although the United Nations Population Fund [15] reported on the working relationship between professional cadres of midwives and physicians, they did not explore the relationship between skilled birth attendants and TTMs. Findings suggest poor relationships exist between midwives and physicians who practice within a hierarchical system where midwives are not respected or trusted. The friction between the two provider groups was shown to inhibit a collaborative practice and, in many cases, resulted in a limited scope of practice for the midwives. For midwives practicing in rural areas with few, if any, obstetricians, midwives were able to practice more autonomously. These findings stimulate further exploration of the relationship between skilled birth attendants and TTMs; in which respect and trust could foster a collaborative practice environment.

### Patient-centered care

In industrialized countries, the concept of patient-centered care has become the hallmark of meeting patients’ needs by allowing their voices to be heard. The concept of patient-centered care can also be implemented in developing countries by exploring patient perspectives and giving them an opportunity to voice their healthcare needs and desires [16]. Past work in developing countries has found patients are more satisfied with healthcare when their providers have a positive attitude, provide the patient with information on their condition and/or treatment, respect the patient, have technical skills, and provide a mechanism for complaints [17,18]. Using a patient-centered approach to care has the ability to improve patient engagement, satisfaction in care received, and adherence to treatment or preventive healthcare recommendations [19]. Patient’s perceptions of their healthcare experience serves as an important quality indicator [16].

For women in developing countries, patient-centered, culturally appropriate care includes maintaining a relationship with their traditionally trained healthcare providers [8,20,21]. As developing countries continue the paradigm shift to a more skilled healthcare workforce to improve maternal and neonatal outcomes, it is important to identify how to effectively incorporate traditional healers and women’s desires into the changing healthcare infrastructure.

Very little data exist on the relationship between skilled birth attendants and TTMs. The concept of a collaborative relationship between a skilled birth attendant and TTM is a relatively new phenomenon with minimal scientific evidence. Successful systems of care depend on the relationships between all providers and patients. Understanding the role of trust and teamwork within the relationship of CMs and TTMs will provide important knowledge to guide future interventions to reduce maternal mortality rates. To examine this changing landscape and to explore the role of TTMs in maternity care, we conducted a pilot study to objectively measure the level of collaboration or trust and teamwork present among TTMs and CMs in Liberia.

## Methods

The trust and teamwork scale was administered as part of a larger United States Agency for International Development funded project- Innovation, Research, Operations, and Planned Evaluation for Mothers and Children (I-ROPE) currently underway. The aim of this operations research is to evaluate the development and implementation of five maternity waiting homes (MWH), connected to rural community clinics in post-conflict Liberia. The five rural clinics with MWHs are matched with five demographically similar clinics without MWHs. An eleventh clinic serving the referral hospital is included in the study to capture those women referred to the next level of care. Traditionally, MWHs have been viewed as a temporary lodging located near a hospital or clinic that is available for women with high risk complications or who live a significant distance from the health center [22]. The MWHs in this study are available for all women irrespective of complications or distance to clinic and are utilized for stays prior to delivery, short and long antenatal stays due to complications (i.e., malaria), and postpartum stays.

Prior to construction of the MWHs, community meetings were held to determine the needs of the communities. Communities agreed the MWHs would be run by a group of TTMs from the catchment area overseen by the CM. Involvement of the TTMs from the outset in the management of the MWH lends the facilities credibility among the community. The long-term goal is to ascertain whether MWHs reduce maternal mortality, increase child survival, and increase the use of skilled midwives during delivery. The study team hypothesized increased communication in the communities with MWHs would impact the trust and teamwork between the TTMs and CMs.

The instrument described in this article utilized a modified version of a pre-existing trust and teamwork scale [23] to evaluate the trust and teamwork among participating TTMs and CMs in the parent study. The instrument was empirically derived through formative research in Ethiopia by Dynes using in-depth interviews with frontline lay health workers including traditional birth attendants [23]. Institutional review board approval was obtained from the University of Michigan, Health Sciences and Behavioral Sciences Review Board and cleared with the Liberian Ministry of Health and Social Welfare.

### Measurement

The *Trust and Teambuilding Scale* was originally developed using semi-structured interviews with 30 healthcare workers in a low resource region of Ethiopia [23]. The concepts of trust and teamwork were examined through participants’ understanding of the attributes, consequences, and conceptualization of the constructs. The original 40–item survey was tested using a four-point Likert scale with “1=Strongly disagree, 2=Disagree, 3=Agree, and 4=Strongly agree” using cards with symbols. Due to the low literacy of the population, participants pointed to a circle indicating “agree” or a square indicating “disagree.” Next, the interviewer opened the folded card to reveal a big circle and a little circle if the participant agreed or a big square and little square if the participant disagreed. The respondent was then questioned as to whether they agreed or disagreed a lot or a little. Use of these response sets with the *Trust and Teambuilding Scale* demonstrated strong internal consistency (Cronbach alpha’s of .79 to .81) and cultural adequacy to assess trust and teamwork in a population of 197 community level health workers in Ethiopia (M. Dynes, Personal Communication, January 30, 2012).

### Sample and setting

Data for this exploratory descriptive study were obtained from a sample of TTMs (n=90) in the 11 rural communities participating in the I-ROPE parent study. The setting for this study is one rural county in north central Liberia with a total population of over 300,000. The population within the catchment communities of the study is 109,000 with 25,000 women of reproductive age.

The official language of Liberia is English. The literacy rate in the adult population is approximately 44% [24]. Inclusion criteria included TTMs providing support to pregnant women in the catchment area of the clinics, participating in the parent study, age 18 or older, and able to speak English or one of the native tribal languages, Kpelle or Mano. All available TTMs participating in the parent study were invited to participate in the study. There were no declinations.

### Survey administration

The study took place between March 2012 and April 2012. The original survey was written in English but included Ethiopian phrases to describe some of the concepts. Prior to survey administration in Liberia, the study team first removed the descriptive Ethiopian phrases from the original survey. When these descriptive Ethiopian phrases were removed, two questions became very similar with both describing the same concept of honesty. To ensure clarity and avoid repeating the same concept, one of these questions was discarded prior to using the survey in Liberia resulting in a total of 39 items.

Two female Liberian study team members, the research nurse for the I-ROPE study and the reproductive health supervisor for the County Health Team, were present for the administration of every survey. They served as the cultural brokers and facilitated translation from English to Kpelle. In addition, in one catchment community, four participants identified Mano as their primary language. At this location a member of the clinic staff (who was not a CM) assisted in translation alongside our approved translators.

Prior to the beginning of data collection, the purpose of the study – to understand trust and teamwork between TTMs and CMs – was explained to participants. Confidentiality was assured and verbal informed consent obtained. Participants were informed they could decline to answer any question and their answers would only be shared in aggregate.

Initially, our team conducted the survey in its original format utilizing individual interviews with TTMs that included one Liberian translator and a female study team member to record the answers that the respondent indicated using the four-point Likert scale with picture cards. However, after piloting this procedure at the first community (n=11), it was determined this process was very lengthy and burdensome to a low-literate population. Additionally, participants had difficulty grasping the meaning of the four-point Likert scale. We assessed their ability to understand ‘agree and disagree’ or ‘yes and no’ using the cards and found dichotomous scoring much easier for comprehension.

In the next community, the research team conducted the survey in a group setting using dichotomous scoring of agree or disagree. Each participant was paired with a female study team member working to record the answers in a private space so others could not see her answers. The questions were read by one of the Liberian translators. Following the question, the participant silently pointed to her response, a circle for agree and a square for disagree, which was then recorded by the research team member.

We also noted the questions written in a ‘double-negative’ format were extremely confusing for the participants. We continued to use the full 39-item *Trust and Teambuilding Scale* in three more communities with a total of 48 participants. The administration of the survey with paired research team members decreased the time commitment of participants; taking approximately 45–60 minutes to complete the full survey. At this point in the study, the research team decided to take a closer look at the questions to determine if the survey could be utilized in an abbreviated version. We decided to make three major changes: 1) eliminate the questions with little to no variability; 2) eliminate any questions written in a double-negative format; and 3) use wording that allowed participants to respond to questions related to their own personal relationship as well as the relationship of other TTMs with CMs in their community. This abbreviated dichotomous version of the instrument, or the *Trust and Teamwork Scale – Liberia,* included eight items to assess trust and ten items to assess teamwork modified from the previous scale.

We were able to use the abbreviated *Trust and Teamwork Scale – Liberia* in six communities with an additional 42 participants. During the administration of the abbreviated version we found the length of time to administer the survey decreased dramatically to approximately 20–30 minutes, participants remained engaged throughout the entire survey, and the dichotomous version was easier for participants to understand. During this process we also noted how the participants responded to the survey. For instance, we determined the translator and participants were describing the responses as ‘truth or lies’ instead of ‘yes or no’ or ‘agree or disagree.’ The participants also found it easier to equate the circle and square on the picture cards to items they were more familiar with; describing the circle as a ‘ball’ and the square as a ‘basket.’

### Procedures for data analysis

For the data analysis, variables were all coded with positive numbers representing more trust and teamwork. New testing of psychometric properties is warranted when a non-standardized measure is changed for subsequent use and is essentially altered into a different measure [25]. This section will report on the reliability and validity of the modified *Trust and Teamwork Scale* for use in Liberia.

Exploratory factor analysis was applied to consolidate items and identify the factors within the *Trust and Teamwork Scale - Liberia*[26]. Due to the dichotomous nature of the observed variables, it was decided to conduct the analysis in Mplus software (Mplus version 6.1) [27]. Mplus is useful because it applies a probit in the place of ordinary least squares, which is important in the analysis of dichotomous variables as a probit does not depend on normal distribution. Because Mplus uses a probit regression of the item on the factor, it allows for analysis of non-linear relationships [28].

Prior to beginning factor analysis the suitability of the sample size was examined. The full survey elicited a total sample size of 42, with an additional 48 completing a portion of the questions in the *Trust and Teamwork Scale – Liberia.* Several questions did not display any variability among participants. Since Mplus is not able to analyze questions without variability, it was determined a priori to exclude these questions from data analysis. This resulted in 16 items for our factor analysis.

Due to our small sample size it was determined missing data would be handled with the default setting in Mplus using all available data to estimate the model [27]. Only one item had missing data, the question “In your village, the CM(s) push their work onto others” was skipped or omitted by one participant in the sample. The literature reports a range of necessary cases per item required for adequate factor analysis ranging from 4 to 10 cases per measure [29-31]. More recently, MacCallum and colleagues [32] took a unique approach to the problem and used factor analytic theory [33] to show it may not be possible to derive a minimum sample size that is appropriate in all situations. Using this decision-making, Guadagnoli and Velicer [34] determined that if components have four or more variables with loadings above .60, then the factor structure or pattern may be interpretable despite the sample size. Exploratory factor analysis with small samples (≤50) have been demonstrated to be a feasible undertaking [35] especially when the data exhibit high loadings, a low number of factors, and high numbers of variables per factor [36,37]. Regardless of the sample size regulations employed, the sample size for this factor analysis was small and limited by the number of available TTMs in our setting. Therefore, a second exploratory factor analysis was employed with a portion of the variables to confirm the initial results.

The construct validity of the *Trust and Teamwork Scale – Liberia* was examined using weighted least squares with mean variance and varimax rotation. In addition to using Mplus for statistical analyses, the Statistical Package for the Social Sciences (SPSS) version 19.0 was utilized for descriptive statistics, reliability, and validity testing. Additional correlational analyses were completed to ascertain the presence of contrast and convergent validity of the revised measure.

## Results

### Participants

Descriptive statistics were conducted to describe the demographic characteristics of the participants. All participants were Liberian TTMs who worked in the 11 catchment communities involved in the parent study. Many of the participants did not know their age, including 26.2% of those who completed the *Trust and Teamwork Scale – Liberia* and 37.5% of those who completed the full scale. The majority of the participants were married with little to no formal schooling. Table 1 compares the demographic characteristics of the 42 participants who completed the *Trust and Teamwork Scale – Liberia* with the 48 participants who completed the full *Trust and Teamwork Scale* on age, marital status, number of children, years of school, years worked as a TTM, days worked per week, and number of deliveries.

**Table 1:** Demographic characteristics % (n)

	**Participants completing full scale**	**Participants completing the Trust and Teamwork Scale – Liberia**	**Total sample**
	**(n=48)**	**(n=42)**	**(n=90)**
**Age range (years)**	21-75	30-67	21-75
Mean (years)	46.5	50.6	48.6
**Marital status**			
Married	72.9 (35)	50.0 (21)	62.2 (56)
Never Married	6.2 (3)	7.1 (3)	6.7 (6)
Divorced	4.2 (2)	7.1 (3)	5.5 (5)
Widowed	16.7 (8)	35.8 (15)	25.6 (23)
**Number of children**	1-19	1-17	1-19
Mean	8.9	7.6	8.3
**Number of living children**	1-19	0-9	0-19
Mean	6.2	4.9	5.6
**Years of formal schooling**			
None	77.1 (37)	69.1 (29)	73.3 (66)
2nd – 6th grade	12.5 (6)	21.4 (9)	16.7 (15)
7th – 12th grade	10.4 (5)	9.5 (4)	10.0 (9)
**Years of experience as a TTM**	< 1 year – 39 years	1 year – 50 years	<1 year – 50 years
Mean (years)	13.6	19.1	16.3

### Construct validity

The 16 items in the *Trust and Teamwork Scale – Liberia* were explored with various exploratory factor analyses using Mplus. Table 2 demonstrates the mean scores, standard deviations, and Kuder-Richardson’s reliability of the scale prior to data analysis.

**Table 2:** ***Trust and Teamwork Scale – Liberia *means and item analysis prior to factor analysis**

	**Completed *Trust and Teamwork Scale – Liberia***			**Full sample**	
	**(n=42)**			**(n=90)**	
**Item**	**M**	**SD**	**α**	**M**	**SD**
**Trust subscale**	6.48	1.63	.629	3.24	.85
In your village, the CM(s) respect you.	.90	.30		.77	.43
In your village, the CM(s) respect all TTMs.	.76	.43			
In your village, the CM(s) have a bond with you.	.79	.42		.89	.32
In your village, the CM(s) have a bond will all the TTMs.	.83	.38			
You have lost confidence in the CM(s) in your village.	.69	.47		.66	.48
The TTMs have lost confidence in the CM(s) in your village.	.67	.48			
You have trust in the CM(s) in your village.	.88	.33		.93	.25
All TTMs have trust in the CM(s) in your village.	.95	.22			
**Item**	**M**	**SD**	**α**	**M**	**SD**
**Teamwork subscale**	7.51	2.01	.701	1.74	.57
In your village, you feel like a part of the team.*	1.0	0			
In your village, do you think all TTMs feel like a part of the team?	.76	.43			
In your village, the CM(s) recognize your experience.*	1.0	0			
In your village, the other TTMs recognize your experience.	.93	.26			
In your village, the CM(s) push their work onto others.	.56	.50			
In your village, the TTMs push their work onto others.	.60	.50			
In your village, the CM(s) and TTMs resolve problems through discussion when you are in disagreement.	.71	.46		.81	.39
In your village, the CM(s) and TTMs are united.	.86	.35		.93	.27
In your village, the CM(s) are an obstacle to your work.	.52	.51			
In your village, the TTMs are an obstacle to your work.	.50	.51			
**Total scale**	14.02	3.37	.810		

* These items were omitted from the reliability analyses and factor analysis due to lack of variability in the n=42 group.

Exploratory factor analysis was performed with dichotomous variables using weighted least squares with mean variance estimator to calculate factor loadings and determine the number of factors that explained correlations among the items. Two rotations were used including an oblique rotation which assumes a correlation between the variables and a varimax rotation which does not assume a correlation between the variables. It was determined to use the results obtained from the varimax rotation due to the low factor correlation in the oblique model (.06).

Using weighted least squares with mean variance estimator and varimax rotation for the 16 variables, we determined the number of factors using the scree plot, eigenvalues, and the conceptual meanings behind the factors. This process indicated a two factor solution with a one factor solution eliciting an eigenvalue of 7.418 and a two factor solution attaining an eigenvalue of 3.007. The elbow of the scree plot was present at two factors. After two factors the eigenvalues dropped to 1.921 and then leveled off. Model fit was measured by using the following goodness of fit measures: 1) a non-significant (p>.05) Chi-Square test of model fit, 2) root mean square error of approximation (RMSEA) of <.05, 3) root mean square residual (RMSR) of <.05, and 4) a Tucker-Lewis index (TLI) of greater than or equal to 0.95 [38,39]. In this analysis, a two factor model was the best fit, supported by the following goodness of fit indicators, where the Chi-Square test of model fit was 84.51(df=89), p=.615; the RMSEA was .000; the RMSR was .165; and the TLI was 1.017.

The two factors elicited from this analysis were named “Trust” and “Teamwork” to match past work done with these items. The rotated matrices for both samples are demonstrated in Table 3.

**Table 3:** **Varimax rotated loadings of a two factor solution for the *Trust and Teamwork Scale – Liberia***

**Item**	**Varimax rotated loadings**		**Varimax rotated loadings**	
	**n=42**		**n=90**	
	Factor 1: Trust	Factor 2: Teamwork	Factor 1: Trust	Factor 2: Teamwork
1. In your village, the CM(s) respect you.*	**.866**	.123	.224	**.551**
2. In your village, the CM(s) respect all TTMs.	**.862**	.330	-	-
3. In your village, the CM(s) have a bond with you.	**.970**	-.085	**.922**	-.021
4. In your village, the CM(s) have a bond will all the TTMs.	**1.008**	.047	-	-
5. You have lost confidence in the CM(s) in your village.	-.101	**.534**	.000	**1.067**
6. The TTMs have lost confidence in the CM(s) in your village.	-.233	**.946**	-	-
7. You have trust in the CM(s) in your village.	.388	.291	.129	-.270
8. All TTMs have trust in the CM(s) in your village.	.351	.377	-	-
9. In your village, do you think all TTMs feel like a part of the team?	**.813**	-.077	-	-
10. In your village, the other TTMs recognize your experience.	.015	**.724**	-	-
11. In your village, the CM(s) push their work onto others.	.617	.502	-	-
12. In your village, the TTMs push their work onto others.	.275	**.725**	-	-
13. In your village, the CM(s) and TTMs resolve problems through discussion when you are in disagreement.	**.800**	-.146	**.828**	.075
14. In your village, the CM(s) and TTMs are united.	**.944**	-.052	**.973**	-.063
15. In your village, the CM(s) are an obstacle to your work.	.652	.518	-	-
16. In your village, the TTMs are an obstacle to your work.	**.623**	.131	-	-

*This variable loaded on different factors in the two analyses.

The item assessing bonding among the CM and all TTMs (item 4) demonstrated high correlations with the other variables, therefore it was removed. Two items also loaded on more than one component at .40 or greater (items 11 & 15) and two items loaded on both components at less than .40 (items 7 & 8) [40]. All five of these items were removed from the final factor structure. Repeat analyses completed with the remaining 11 items that loaded on a factor at >.40 demonstrated a good model fit with a two factor solution as evidenced by a Chi-Square test of model fit, 32.03(df=34), p=.564; a RMSEA of .000; a RMSR of .140; and a TLI of 1.014. The Chi-Square test of difference, 31.38(df=10), p<.001, also indicated that a two-factor structure provided the better fit. The communalities describing the amount of variance the factors explain in each variable are presented in Table 4 for the final factor structure.

**Table 4:** **Communalities for *Trust and Teamwork Scale - Liberia *(n=42)**

** Variable**	**Communalities**
**Trust subscale**	
1. In your village, the CM(s) respect you.	.737
2. In your village, the CM(s) respect all TTMs.	.823
3. In your village, the CM(s) have a bond with you.	.996
9. In your village, do you think all TTMs feel like a part of the team?	.691
13. In your village, the CM(s) and TTMs resolve problems through discussion when you are in disagreement.	.627
14. In your village, the CM(s) and TTMs are united.	.906
16. In your village, the TTMs are an obstacle to your work.	.369
**Teamwork subscale**	
5. You have lost confidence in the CM(s) in your village.	.309
6. The TTMs have lost confidence in the CM(s) in your village.	.914
10. In your village, the other TTMs recognize your experience.	.346
12. In your village, the TTMs push their work onto others.	.783

The exploratory factor analysis was repeated with weighted least squares with mean variance estimator and varimax rotation using a sample size of 90 (including the 42 used in the original analyses). Unfortunately, all members of this sample did not complete all 16 items of the *Trust and Teamwork Scale – Liberia*, so this analysis was completed with the six items as demonstrated in Table 3. The scree plot and eigenvalues for these six items again indicated a two factor solution with a one factor solution eliciting an eigenvalue of 2.736 and a two factor solution attaining an eigenvalue of 1.706. A two factor model was supported as the best fit by a Chi-Square test of model fit, 1.44(df=4), p=.837; a RMSEA of .000; a RMSR of .057; and a TLI of 1.078. The Chi-Square test of difference, 14.33(df=5), p<.05, continued to support the two-factor structure as a better fit. A summary of the fit indices for the final variables in both samples can be seen in Table 5. The two factors resulting from this repeat analysis matched the past factors of “Trust” and “Teamwork,” with one item loading on a different factor during the repeat analysis. The item, “In your village, the CM(s) respect you” loaded on the “Trust” factor during the first analysis with n=42 and loaded on the “Teamwork” factor during the second analysis with n=90.

**Table 5:** **Fit indices for *Trust and Teamwork Scale – Liberia *final items (n=11 questions)**

	**n=42**		**n=90**	
	**1 factor model**	**2 factor model**	**1 factor model**	**2 factor model**
**Chi-Square Test of Model Fit**	69.94(df=44), p=.0077	32.03(df=34), p=.5644	21.11(df=9), p=.0122	1.44(df=4), p=.8372
**RMSEA**	.118	.000	.122	.000
**RMSR**	.229	.140	.202	.057
**TLI**	.861	1.014	.836	1.078
**Chi-Square Test of Difference**	31.38(df=10), p<.001		14.33(df=5), p<.05	

In summary, exploratory factor analysis indicated the new 11-item scale containing two components was theoretically congruent with the concepts of trust and teamwork among TTMs in rural Liberia. Of the newly derived components, factor 1, “Trust,” contains seven items, and factor 2, “Teamwork,” contains four items.

### Reliability

Before conducting factor analysis, the internal consistency of the *Trust and Teamwork Scale – Liberia* was assessed using Kuder-Richardson's alpha, which measures the alpha coefficient for dichotomous variables. Nunnally [31] reports the reliability coefficient should be .70 or greater for a new instrument and .80 or greater for an instrument that has previously been tested. The Kuder-Richardson’s alpha coefficient for the full scale was .810, the trust subscale was .629, and the teamwork subscale was .681. The reliabilities of the new scale were assessed after factor analysis at .717 for the full scale, .824 for the trust subscale, and .621 for the teamwork subscale. Pallant [40] notes it is not uncommon for short scales, or those with fewer items, to have lower reliability values, therefore we consider our reliability values within range for a newly developed scale (Table 6).

**Table 6:** Mean, standard deviation, and internal reliabilities for the two newly derived subscales (n=42)

**New subscale**	**M**	**SD**	**α**
**Factor 1: Trust**	5.29	2.04	.824
(items 1, 2, 3, 9, 13, 14, 16)			
**Factor 2: Teamwork**	2.88	1.19	.621
(items 5, 6, 10, 12)			

### Contrast validity

Contrast validity is used to assess the differences measured by an instrument between two diverse groups. Contrast validity was determined using an independent *t*-test between TTMs in communities where MWHs had been built and TTMs in communities where MWHs had not been built. The hypothesis being tested for contrast validity was: “When TTMs from the intervention group (MWHs) are compared with the TTMs from the comparison group (no MWH); the intervention group will have higher trust and teamwork scores.” As hypothesized, among the sample of 42 TTMs who completed the full *Trust and Teamwork Scale - Liberia* the *t*-test revealed significant differences (p=.002) between trust among the TTMs in communities with maternity waiting homes and those without maternity waiting homes, however no significant differences were noted in teamwork between the two groups (see Table 7).

**Table 7:** Mean comparison between TTMs with and without maternity waiting homes in their villages (n=42)

**Factor**	**MWH Present (n=7)**		**No MWH (n=35)**				
	**M**	**SD**	**M**	**SD**	**t**	**df**	**p**
**Factor 1. Trust**	6.43	.53	5.06	2.15	−3.29	37.84	.002
(items 1, 2, 3, 9, 13, 14, 16)							
**Factor 2. Teamwork**	3.29	1.11	2.80	1.21	.98	40	.332
(items 5, 6, 10, 12)							

Among the full sample of participants (n=90) the *t*-test indicated a significant difference (p=.001) in trust between TTMs and CMs in communities with and without MWHs with maternity waiting home communities reporting higher levels of trust. Significant differences were noted in teamwork (p=.024) with TTMs in communities without MWHs reporting higher levels of teamwork (see Table 8). The results of the Teamwork comparison were the opposite of what was hypothesized among the full sample of participants. This discrepancy may be related to: 1) the change in factor loadings for the item “In your village, the CM(s) respect you.” or 2) the fact that the full sample did not complete the entire scale.

**Table 8:** Mean comparison between TTMs with and without maternity waiting homes in their villages (n=90)

** Factor**	**MWH Present (n=45)**		**No MWH**				
			**(n=35 for trust analyses, n=45 for teamwork analyses)**				
	**M**	**SD**	**M**	**SD**	**t**	**df**	**p**
**Factor 1. Trust**	2.91	.29	2.23	1.09	−3.62	37.72	.001
(items 3, 13, 14)							
**Factor 2. Teamwork** (items 1, 6)	1.24	.88	1.60	.54	2.31	72.82	.024

### Convergent validity

A scale is able to demonstrate convergent validity when it establishes a high correlation with another item or scale that measures the same construct [26]*.* Convergent validity was tested using each factor independently and correlating it with a related item from the study. Unfortunately, there were limited items assessed in this study and we were only able to test the convergent validity of the trust subscale. The correlation between the two trust questions (“You have trust in the CMs in your village” & “All TTMs have trust in the CMs in your village”) and the trust subscale (including all seven items: 1, 2, 3, 9, 13, 14, 16) was assessed. A small correlation approaching significance (r=.285, p=.067) indicated that with a larger sample size the trust subscale may be correlated with the questions about the individual and other TTMs having trust of the CMs in their respective villages. Table 9 displays the correlations among the sample (n=42) that completed all of the final items in *Trust and Teamwork Scale – Liberia*.

**Table 9:** **Correlation matrix of retained questions within the *Trust and Teamwork Scale – Liberia *(n=42)**

		**Trust**							**Teamwork**			
		**The CMs respect you.**	**The CMs respect all TTMs**	**The CMs have a bond with you.**	**Do you think all TTMs feel like a part of the team.**	**The CMs & TTMs resolve problems through discussion when in disagreement.**	**The CMs & TTMs are united.**	**The TTMs are an obstacle to your work.**	**You have lost confidence in the CM(s) in your village.**	**The TTMs have lost confidence in the CM(s) in your village.**	**The other TTMs recognize your experience.**	**The TTMs push their work on to others.**
**Trust**	The CMs respect you.	1.0										
	The CMs respect all TTMs	.768	1.0									
	The CMs have a bond with you.	.839	.841	1.0								
	Do you think all TTMs feel like a part of the team.	.699	.702	.830	1.0							
	The CMs & TTMs resolve problems through discussion when in disagreement	.635	.618	.780	.650	1.0						
	The CMs & TTMs are united.	.782	.772	.946	.788	.752	1.0					
	The TTMs are an obstacle to your work.	.522	.546	.590	.492	.445	.569	1.0				
**Teamwork**	You have lost confidence in the CM(s) in your village.	−.006	.079	−.121	−.099	−.162	−.160	.005	1.0			
	The TTMs have lost confidence in the CM(s) in your village.	−.032	.113	−.233	−.191	−.297	−.298	−.007	.531	1.0		
	The other TTMs recognize your experience.	−.076	.168	−.033	−.025	−.099	−.080	.064	.323	.552	1.0	
	The TTMs push their work on to others.	.399	.531	.299	.252	.131	.215	−.007	.415	.702	.479	1.0

## Discussion

Psychometric testing of the *Trust and Teamwork Scale - Liberia* determined that only 11 of the original 18 items should remain in the scale. This exploratory factor analysis used Mplus for dichotomous variables to elicit a two-factor solution utilizing weighted least squares with mean variance estimator and varimax rotation. The two-factor solution provided preliminary evidence that the “Trust” and “Teamwork” factors may be attributed to increased communication and cooperation in constructing MWHs within Liberian communities. The final two-factor solution demonstrated a good factor structure with a Chi-Square test of model fit, 32.03(df=34), p=.564; a RMSEA of .000; a RMSR of .104; and a TLI of 1.014. The reliabilities of the new factors ranged from .621 to .824, which are acceptable for a new instrument. A portion of the *Trust and Teamwork Scale-Liberia* also demonstrated an adequate two-factor structure in repeat analyses in the larger sample size with a Chi-Square test of model fit, 1.44(df=4), p=.837; a RMSEA of .000; a RMSR of .057; and a TLI of 1.078. Repeat analyses with the larger sample size supported the general factor structure except for a change in factor loadings of one item, “In your village, the CM(s) respect you.”

### Lessons learned

Bulmer and Warwick [41] note there are difficulties in conducting survey research in developing countries which can include problems with recruitment, language and translation, interviewer training, and situational variables which include country specific structural and cultural variables. Our study demonstrated that even with an established research team in the country, these situational and cultural variables may still arise. One of our biggest initial challenges was the language and translation aspect of this study. We retained the questions as written in the original *Trust and Teambuilding Scale*, but found it necessary to ensure conceptual equivalence of translated items, not literal translation [41]. In order for our participants to grasp the concepts being discussed it was necessary for the survey items to be in a context they understood, including using the answers of ‘truth and lies’ instead of ‘yes and no.’

An additional obstacle encountered during the survey administration was the use of Likert scale answer keys. Past work published in the transportation literature has reported it is difficult to use rating scales in developing countries but short verbal rating scales have proven to be the most valid and reliable approach [42]. However, our study demonstrated that conducting research with short Likert scales in rural Liberia did not prove feasible. Heine and colleagues [43] note Likert scales do not provide a context-free answer because they capture an individual’s response in relation to a shared norm. In order to alleviate this problem, it is recommended that more objective questions be used [43]. However, when the basis of the study is to identify subjective attitudes, cross-cultural comparisons, or even cross-cultural use of a measurement tool, it may prove very difficult due to changes in the reference group [43]. This effect was demonstrated in our study.

The current body of literature is limited on best practices for survey administration and the use of established measures with non- and low-literate populations. Despite the difficulties encountered during the survey administration process, our research team learned valuable lessons. We captured important cultural considerations we could not have predicted. This process reinforced the need to pilot test tools and to be flexible to the needs of study populations.

### Limitations

We acknowledge the sample size of this study was small and the *Trust and Teambuilding Scale* is still in its infancy. However, the process of using a pre-existing tool in a new cultural situation cannot be overemphasized. This process reinforced the need for additional work and research into conducting surveys in non- and low-literate populations and the importance of evaluating tools for cross-cultural use. Additionally, our work supports cross-cultural validation of tools is essential. Our survey was administered in a setting where each respondent worked with at least two members of the research team including a Liberian translator and a female member of the research team. Although this was unavoidable due to the need to have a translator and someone to record the answers for the non- and low-literate population, this methodology may have introduced a power dynamic that had the potential to influence a social desirability bias. Future work should continue to evaluate the use of tools across cultures. Additional work is needed to confirm the factor structure of the *Trust and Teamwork Scale – Liberia.*

## Conclusion

The *Trust and Teamwork Scale – Liberia* demonstrated the ability of the tool to measure trust and teamwork in a sample of mostly non-literate TTMs in rural Liberia. As we continue to strengthen health systems to improve maternal health in developing countries, it is important to consider the role of traditional healthcare providers, such as TTMs, and how they can be integrated into the changing healthcare structure. Evaluating collaboration across cadres of healthcare workers using measures such as trust and teamwork may be one way to assess the relationship among traditional health workers and skilled health professionals.

In Liberia, TTMs play an important role in the healthcare system by serving as a support system and reservoir of knowledge related to pregnancy and maternal health. Women in Liberia continue to utilize TTMs for their healthcare needs based on convenience and choice. A culturally sensitive approach to reducing maternal mortality must consider a patient-centered approach and acknowledge the important role TTMs play in women’s lives. Trust and teamwork between the TTM and CM ensure that evidence based care is being delivered by the TTM, which has the potential to improve maternal health outcomes. The *Trust and Teamwork Scale – Liberia* provides a tool to begin to explore the clinical implications of skilled birth attendants and TTMs working together to improve maternal health outcomes. Future work is underway to confirm the factor structure of the *Trust and Teamwork Scale – Liberia* with a larger population.

## Abbreviations

CM: Certified midwives; I-ROPE: Innovation, Research, Operations, and Planned Evaluation for Mothers and Children; MWH: Maternity waiting home; TTMs: Trained traditional midwives.

## Competing interests

The authors declare that they have no competing interests.

## Authors’ contributions

JL conceived of the study, participated in its design and coordination, carried out data collection, performed statistical analysis, and was involved in drafting and revising the manuscript. MM participated in the study design and coordination, carried out data collection, performed statistical analysis, and was involved in drafting and revising the manuscript. JM participated in the acquisition of data and drafting and revising the manuscript. JF was involved in study design and revising the manuscript. All authors read and approved the final manuscript.

## Pre-publication history

The pre-publication history for this paper can be accessed here:

http://www.biomedcentral.com/1472-6963/13/134/prepub
